# The Impact of a Multidisciplinary Patient Education Program on Venous Leg Ulcer Healing: A Randomised Controlled Trial

**DOI:** 10.1111/wrr.70084

**Published:** 2025-08-29

**Authors:** Sebastian Probst, Camille Saini, Paul Bobbink, André Frei, Fiona Dudley‐Martin, Simone Gafner, Florian Stern, Georgina Gethin

**Affiliations:** ^1^ Geneva School of Health Sciences HES‐SO University of Applied Sciences and Arts, Western Switzerland Geneva Switzerland; ^2^ Care Directorate, Geneva University Hospitals Geneva Switzerland; ^3^ Faculty of Medicine University of Geneva Geneva Switzerland; ^4^ Faculty of Medicine Nursing and Health Sciences Monash University Clayton Australia; ^5^ School of Nursing and Midwifery University of Galway Galway Ireland; ^6^ Institute of Health Sciences, Department of Physiotherapy, School of Health Sciences HES‐SO Valais‐Wallis Leukerbad Switzerland

**Keywords:** compression therapy, patient education, self‐management, venous leg ulcers, wound healing

## Abstract

To evaluate the impact of a nurse‐led, multidisciplinary education programme on wound healing, patient knowledge, and adherence to compression therapy, physical activity, and nutrition in individuals with venous leg ulcers (VLU). In this randomised controlled trial, 87 patients with VLU from three outpatient clinics in Western Switzerland were allocated to an intervention group (IG) receiving structured education plus standard care, or a control group (CG) receiving standard care alone. The 12‐month intervention included in‐person education, counselling, and follow‐ups. The primary outcome was complete wound closure at 12 months. Secondary outcomes included wound area reduction, patient knowledge, adherence behaviours, and ulcer recurrence. At 12 months there was no significant difference in complete wound closure between groups (*p* = 0.668). Wound area reduction was significantly greater in the IG at 1 month (54.0% vs. 35.6%, *p* = 0.041). The IG showed earlier and greater improvements in knowledge, self‐efficacy, and adherence to compression therapy and mobility. No significant differences in nutritional behaviour or body weight were observed. Nurse‐led education improved early healing and patient engagement. Sustained behaviour change may require longer‐term support and targeted nutritional interventions. Future research should explore adaptive education models and digital tools for long‐term VLU management.

## 1 Introduction

Venous leg ulcers (VLU) are a chronic and debilitating condition, primarily affecting older adults with chronic venous insufficiency. These ulcers significantly impact patient quality of life, leading to pain, restricted mobility, and increased healthcare utilisation, while also posing a substantial financial burden on healthcare systems [1, 2, 3]. The global prevalence of VLU is estimated at 0.32% of the population [4], with these wounds accounting for 60% of all chronic ulcers, making their management a considerable challenge [5]. Despite advancements in compression therapy and wound care, healing rates remain suboptimal, with only 42.2% of ulcers closing within 3 months and 48.6% within 6 months [6]. Moreover, recurrence rates are high, with up to 70% of patients developing new ulcers within 6 months of initial healing [7].

Effective VLU management requires a comprehensive, multidisciplinary approach, integrating wound care, compression therapy, physical activity, leg elevation, and nutritional optimisation [8, 9, 10]. However, adherence to these essential protocols remains inconsistent, with compression therapy compliance ranging from 10% to 80%, significantly impacting healing outcomes and recurrence prevention [11]. Studies indicate that patient education is a key determinant of adherence, as greater knowledge and self‐management skills improve compliance and clinical outcomes [11, 12, 13, 14, 15].

Nurses play a central role in VLU care, often serving as the primary point of contact for patients throughout treatment [15]. Nurse‐led interventions, which incorporate structured patient education, adherence monitoring, and individualised wound care strategies, have demonstrated promising effects on healing outcomes [10, 14]. However, despite these benefits, further research is needed to establish the long‐term effectiveness of such interventions, particularly among high‐risk populations, where chronic wounds have a greater likelihood of delayed healing and recurrence.

In response to these challenges, this study investigates the impact of a nurse‐led multidisciplinary patient education programme designed to enhance patient knowledge and promote adherence to evidence‐based VLU management strategies. The intervention integrates comprehensive education on ulcer aetiology, compression therapy, mobility exercises, leg elevation, and nutrition, aiming to empower patients and improve self‐management behaviours.

The primary objective of this study was to evaluate the effectiveness of the nurse‐led patient education programme on wound healing at 12 months, specifically ulcer closure rates and size reduction. Secondary objectives were to evaluate healing outcomes and size reduction at 1 month and 3 months and the impact on patient knowledge, adherence to compression therapy, mobility engagement, and nutritional behaviour at 12 months.

## 2 Materials and Methods

### 2.1 Trial Design

A multicenter, randomised controlled trial was registered under NCT04019340 and conducted in three wound care outpatient clinics in Western Switzerland. Ethical approval was granted by the Geneva Ethics Committee (2019‐01964).

### 2.2 Participants and Sample Size

Participants meeting the following criteria were eligible for inclusion: aged 18 years or older, having an existing open VLU or VLU associated with non‐severe peripheral arterial disease (PAD), a wound surface area ≥ 2 cm^2^, and proficient in the French language. The diagnosis of VLU was determined by the attending physician. Participants were excluded if they were unable to provide valid informed consent.

The sample size calculation was based on the assumption that 30% of patients with VLU or VLU associated with non‐severe PAD achieve complete wound closure within 3 months under standard of care [16, 17]. We aimed to detect a minimum difference of 20% in the proportion of wound closure at 3 months between the control group (30%) and the intervention group (50%). This target difference is consistent with findings from similar studies focusing on educational interventions [14]. With a one‐sided significance level of 0.05% and 80% power, the required sample size was *n* = 148, with participants randomised in a 1:1 ratio. To account for an anticipated 20% attrition rate, it was estimated that *n* = 185 patients would need to be screened to achieve the desired sample size.

### 2.3 Intervention

Participants in the CG received standard wound care according to the outpatient clinic; participants in the IG received an additional educational intervention.

Standard of care was defined as the routine outpatient wound management provided by the clinic, including wound assessment, dressing application, and compression therapy, delivered in accordance with institutional clinical practice guidelines and without additional structured educational support.

The educational intervention, developed by nurses, physiotherapists and dieticians [18, 19], was structured into two main components: nurse‐led interactive sessions and a brochure.

**FIGURE 1:**
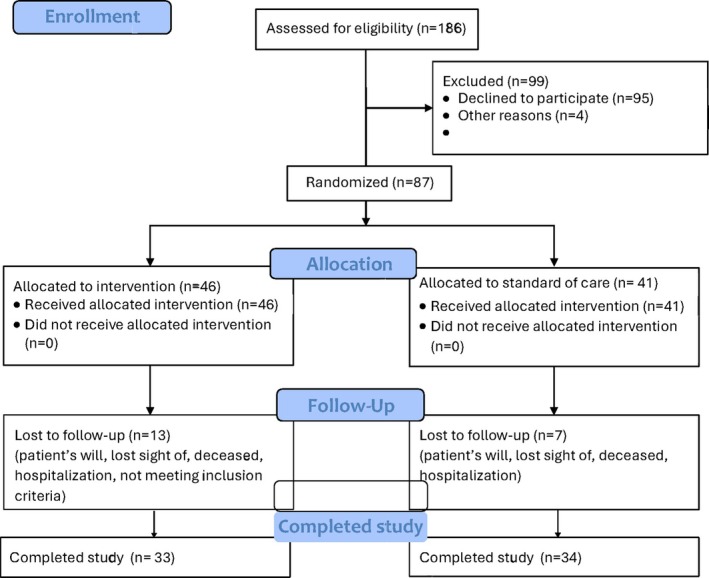
Participants flow diagram.

After randomization, participants allocated to the IG received the brochure and were asked to read it before the next visit. Over the first 5 weeks of post‐enrollment, they attended weekly one‐hour education sessions. These education sessions took place at the outpatient clinic or at the participant's home and covered essential topics such as the aetiology and pathophysiology of VLU, proper use of compression stockings, lower extremity exercises, physical activity, leg elevation, trauma prevention, proper skin care, and the importance of a protein‐rich diet. The brochure was used as educative support throughout the sessions. It also represented a reliable source of information that participants could consult at home and that could allow them to pursue their reflections and/or record their questions. Any questions relating to the brochure were answered by the nurse during the next sessions. This phase was followed by five additional counselling sessions at weeks 6, 9, and 12, focusing on self‐management, self‐efficacy, and addressing patient‐specific concerns (see study flow chart Figure 1). These telephone counselling sessions aimed to reinforce therapeutic adherence and to strengthen the self‐belief necessary for effective, sustained self‐management. All educational activities were conducted by wound care expert study nurses using a standardised protocol. The study nurses were trained before commencement of the study, and regular meetings were performed to ensure consistency.

### 2.4 Data Collection and Outcomes

The primary outcome was the proportion of wounds closed at 12 months. Wound closure was defined as complete skin epithelialisation or re‐epithelialisation without drainage of the wound surface confirmed by the wound care study nurse and by one picture, independently verified by a dermatologist blinded to group allocation.

Secondary outcomes included complete wound closure after one, 3, 6, and 12 months, as well as other indicators of ulcer healing such as percentage wound area reduction (PWAR) at one, 3, 6, and 12 months, PWAR ≥ 40% at 1 month as a predictor of complete wound closure at 3 months [20], and the probability of wound closure over the 12‐month period. In addition, the following outcomes were assessed at baseline, 1, 3, 6, and 12 months. They included a score of patient knowledge and self‐efficacy using the validated VeLUSET_FR questionnaire [21, 22]. Effective physical activity levels were monitored with GeneActiv accelerometers during 1 week starting at each of the five timepoint visits [23]. Adherence to prescribed lifestyle modifications, such as physical activity, leg elevation, ankle exercises, and compression stockings use, was measured via a health questionnaire. Nutritional status was assessed using the Mini Nutritional Assessment (MNA) questionnaire [24, 25], daily protein intake was estimated through the Frequent Food Questionnaire (FFQ) [26], and the presence/absence of overweight was assessed by calculating the body mass index (BMI). The wound evaluation was reported at baseline and covered exudative status, maceration, odour, and pain. In addition, ulcer recurrence was documented throughout the study and defined as the development of a new ulcer in participants with a history of VLU.

If a patient had more than one ulcer, the largest ulcer was deemed to be the reference ulcer.

Data was collected in the same way in the IG and CG by each group's own trained study nurse at baseline [*T*_0_], at month 1, 3, 6, and 12 months. To ensure accurate completion and understanding of the different instruments, data entry was performed by the study nurses jointly with the participants in an electronic case report form. At each data collection timepoint, the wound was photographed and its dimensions (width, length, area) measured with an imaging device (eKare) to ensure reproducibility [17] after opening the dressing and wound cleansing. Standardisation of the assessment was achieved by training of study nurses and by using the imaging device [27]. All wound tracings were subsequently verified by a wound expert blinded to group allocation at the end of data collection. A random sample of a set of pictures of 5% of participants was checked by a dermatologist blinded to group allocation.

### 2.5 Assignment of Intervention

Participants were randomly assigned to the IG or CG after baseline data collection using a computer‐generated 1:1 randomisation list. Allocation was concealed through grey sealed opaque serially numbered envelopes, which were opened by the study nurse to determine group assignment. Due to the educational nature of the intervention, blinding was not feasible for participants or staff. However, the wound expert and the dermatologist verifying wound contours, as well as the statistician analysing the data, remained blinded to group assignments.

### 2.6 Statistical Plan

The statistical analyses were performed using STATA 17.0 and R 4.4.0 software by a statistician blinded to treatment allocation. Descriptive statistics were used to summarise demographic and health data for both IG and CG, with categorical variables expressed as frequencies and percentages, and continuous variables as means with standard deviations [SD] or medians with interquartile ranges [IQR], depending on data distribution.

Comparative statistics were used regarding the outcomes. For the primary outcome, the proportion of complete wound closure was compared between groups using Chi‐Square or Fisher's exact tests. Secondary outcomes were analysed similarly, with continuous variables compared between groups using *t*‐tests or Wilcoxon rank‐sum non‐parametric tests, depending on data distribution, and binary outcomes evaluated using Chi‐Square or Fisher's exact tests. Longitudinal intra‐group variations were assessed using paired *t*‐tests or Wilcoxon signed‐rank test for continuous parametric or non‐parametric variables, respectively, and using McNemar test for categorical variables. The probability of wound closure at 3 months was analysed between treatment groups using the Kaplan–Meier test followed by the log‐rank test.

To maximise power, missing data were handled by multiple imputations by chained equations (MICE) and all analysis was performed on the complete convenience sample of *n* = 87.

## 3 Results

A total of 186 patients were assessed for eligibility between February 2020 and May 2024. Recruitment was severely limited due to the onset of the COVID‐19 pandemic, and a higher‐than‐expected refusal of participation occurred. Consequently, we proceeded with a convenience sample of *n* = 87, randomly allocated to either the intervention group (IG) (*n* = 46) or the control group (CG) (*n* = 41). Of the 87 participants, 67 completed all trial visits, representing an attrition rate of 23% (28.3% in IG (*n* = 13/46) and 17.1% in CG (*n* = 7/41)) (Figure 1). Attrition rates were mainly due to loss to follow‐up, patients' will, hospitalisation, or death (Figure 1).

### 3.1 Participant Characteristics

The study included slightly more men (54%, *n* = 47) than women (46%, *n* = 40), with a mean age of 68.2 years (SD 14.6) and a mean BMI of 32.2 (SD 9.0). Both groups were similar regarding VLU‐related, health‐related, and socio‐demographic characteristics (Table 1). Median ulcer area was 6.9 cm^2^ (IQR 3.5–16.7) in the IG and 5.9 cm^2^ (IQR 3.3–17.5) in the CG. In the majority of cases, these ulcers were recurrent wounds (65.2%, *n* = 30/46, in IG and 61%, *n* = 25/41, in CG). Of note, more participants wore compression stockings in the CG (39%, *n* = 16/41) than in the IG (19.6%, *n* = 9/46) (*p* = 0.059), and conversely, more participants wore compression bandages in the IG (91.3%, *n* = 42/46) than in the CG (73.2%, *n* = 30/41) (*p* = 0.044).

**TABLE 1:** Participants characteristics.

Sociodemographics	Intervention (*n* = 46)	Control (*n* = 41)	Total (*n* = 87)
Age, mean (SD)	67.0 (14.4)	69.6 (14.8)	68.2 (14.6)
Sex, *n* (%)			
Female	22 (47.8)	18 (43.9)	40 (46.0)
Male	24 (52.2)	23 (56.1)	47 (54.0)
Civility, *n* (%)			
Single	7 (15.2)	7 (17.1)	14 (16.1)
Married	15 (32.6)	19 (46.3)	34 (39.1)
Divorced	14 (30.4)	8 (19.5)	22 (25.3)
Widowed	10 (21.7)	7 (17.1)	17 (19.5)
Education, *n* (%)			
Mandatory school	9 (19.6)	7 (17.1)	16 (18.4)
Maturity or equivalent	6 (13.0)	7 (17.1)	13 (14.9)
Credentialed professional	31 (67.4)	27 (65.8)	58 (66.7)
Occupation, *n* (%)			
Active	13 (28.3)	10 (24.4)	23 (26.4)
Retired	26 (56.5)	25 (61.0)	51 (58.6)
Disability insurance beneficiary	2 (4.4)	5 (12.2)	7 (8.1)
Other	5 (10.9)	1 (2.4)	6 (6.9)
Annual salary, *n* (%)			
< 25,000 CHF	4 (8.7)	8 (19.5)	12 (13.8)
25,001–50,000 CHF	17 (37.0)	17 (41.5)	34 (39.1)
50,001‐100,000 CHF	16 (34.8)	9 (22.0)	25 (28.7)
> 100,000 CHF	3 (6.5)	3 (7.3)	6 (6.9)
Does not want to answer	6 (13.0)	4 (9.8)	10 (11.5)

VLU‐related information	Intervention	Control	Total
Area (cm^2^), median (IQR)	6.9 (3.5–16.7)	5.9 (3.3–17.5)	6.5 (3.3‐17.5)
Participants with a first VLU, *n* (%)	16 (34.8)	16 (39.0)	32 (36.8)
Participants with a recurrent VLU, *n* (%)	30 (65.2)	25 (61.0)	55 (63.2)
Number of recurrence, mean (SD) (min–max)	3.0 (3.4) (1–15)	3.6 (3.7) (1–15)	3.3 (3.5) (1–15)
Hospitalised once because of this VLU, *n* (%)	16 (34.8)	17 (41.5)	33 (37.9)
Number of days, mean (SD) (min–max)	20.3 (27.9) (4–120)	24.8 (41.8) (5–180)	22.6 (35.2) (4–180)
Exudate, *n* (%)	44 (95.7)	36 (87.8)	80 (92.0)
Maceration, *n* (%)	20 (43.5)	16 (39.0)	36 (41.4)
Oedema, *n* (%)	32 (69.6)	34 (82.9)	66 (75.9)
Odour, *n* (%)	11 (23.9)	14 (34.1)	25 (28.7)
Odour intensity (VAS 0‐100), mean (SD) (min–max)	28.8 (31.4) (4–90)	35.6 (26.2) (4–75)	32.5 (28.3) (4–90)
Pain, *n* (%)	32 (69.6)	32 (78.0)	64 (73.6)
Pain intensity (VAS 0‐10), mean (SD) (min–max)	4.2 (2.1) (2–10)	4.2 (2.3) (1–10)	4.2 (2.2) (1–10)
Physical activity, *n* (%)	40 (87.0)	35 (85.4)	75 (86.2)
Effective activity (GeneActiv)			
Sleep (h), mean (SD)	5.5 (2.1)	4.9 (2.1)	5.2 (2.1)
Sedentary (h), mean (SD)	13.7 (2.6)	13.1 (3.5)	13.4 (3.1)
Light (h), mean (SD)	3.8 (3.2)	4.1 (3.4)	3.9 (3.3)
Moderate (min), mean (SD)	81.0 (75.0)	106.0 (85.9)	92.7 (80.8)
Vigorous (min), mean (SD)	0.1 (0.4)	0.3 (1.0)	0.2 (0.8)

Other health information	Intervention	Control	Total
BMI, mean (SD)	31.7 (9.0)	32.8 (9.0)	32.2 (9.0)
MNA score, *n* (%)			
Normal	38 (82.6)	31 (75.6)	69 (79.3)
At risk	8 (17.4)	10 (24.4)	18 (20.7)
Smoker, *n* (%)	11 (23.9)	12 (29.3)	23 (26.4)
Pack per week, mean (SD) (min–max)	5.7 (1.9)	5.8 (2.2)	5.8 (2.0)
Diabetes, *n* (%)	14 (30.4)	8 (19.5)	22 (25.3)
Compression bandage, *n* (%)	42 (91.3)	30 (73.2)	71 (81.6)
Compression stockings, *n* (%)	9 (19.6)	16 (39.0)	25 (28.7)
Leg elevation, *n* (%)	37 (80.4)	39 (95.1)	76 (87.4)
Ankle exercise, *n* (%)	28 (60.9)	17 (41.5)	45 (51.7)
Physical activity, *n* (%)	40 (87.0)	35 (85.4)	75 (86.2)
Effective activity (GeneActiv)			
Sleep (h), mean (SD)	5.5 (2.1)	4.9 (2.1)	5.2 (2.1)
Sedentary (h), mean (SD)	13.7 (2.6)	13.1 (3.5)	13.4 (3.1)
Light (h), mean (SD)	3.8 (3.2)	4.1 (3.4)	3.9 (3.3)
Moderate (min), mean (SD)	81.0 (75.0)	106.0 (85.9)	92.7 (80.8)
Vigorous (min), mean (SD)	0.1 (0.4)	0.3 (1.0)	0.2 (0.8)

### 3.2 Impact of the Intervention on Ulcer Healing

#### 3.2.1 Complete Wound Closure

**FIGURE 2:**
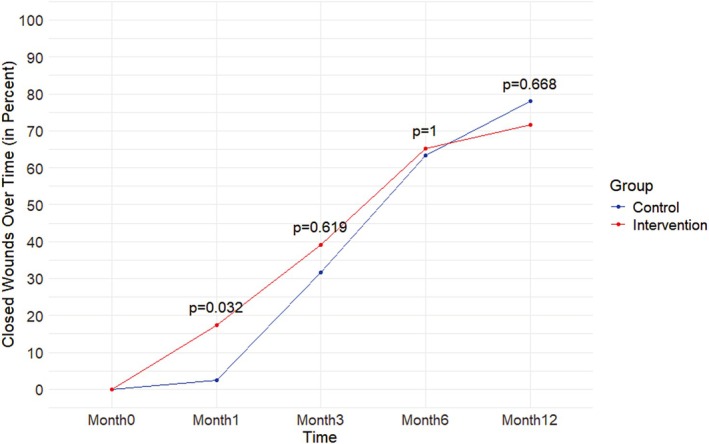
Wound closure over time.

For the primary outcome, at 12 months there was no significant difference in wound closure between groups (*p* = 0.668). Notably, at 3 months, 39.1% (*n* = 18/46) in the IG were completely healed versus 31.7% (*n* = 13/41) in the CG (*p* = 0.508). This effect was most pronounced at the beginning of the 3‐month treatment period, i.e., at 1 month from commencement of the study, when 17.4% (*n* = 8/46) of the wounds were completely healed in the IG, versus 2.4% (*n* = 1/41) in the CG, a 15% higher proportion of healed wounds in the IG (Fisher exact, *p* = 0.032) (Figure 2).

#### 3.2.2 Percentage Wound Area Reduction (PWAR)

**FIGURE 3:**
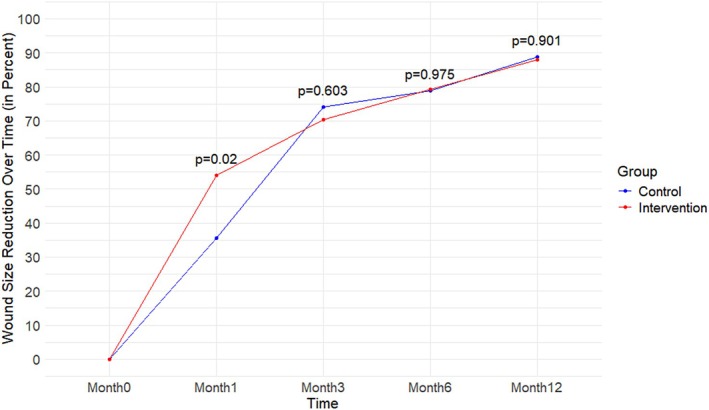
Percentage wound area reduction over time.

Mean PWAR was higher in the IG than in the CG at the earliest time point of 1 month. The IG experienced a 54% mean PWAR after 1 month, versus the CG's 35.6% mean PWAR, resulting in an 18.4% greater reduction in the IG (*p* = 0.020). At later timepoints, mean PWAR was similar in both groups (Figure 3).

The PWAR ≥ 40% at month one was significantly higher in the IG (67.4%, *n* = 31/46) than in the CG (46.3%, *n* = 19/41) (*p* = 0.047).

#### 3.2.3 VLU Recurrence

Recurrence was rather low in the cohort and no significant difference was observed between the two groups during the study (17.4%, *n* = 8/46, in the IG and 7.32%, *n* = 3/41, in the CG, *p* = 0.054).

### 3.3 Impact of the Intervention on Knowledge and Health Behaviour

#### 3.3.1 Knowledge About VLU and Self‐Efficacy

**FIGURE 4:**
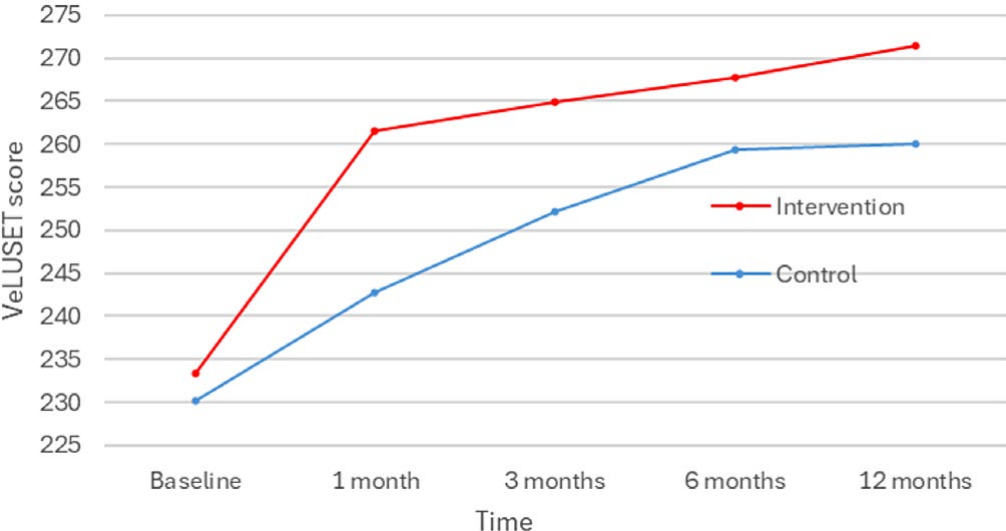
Knowledge about VLU and self‐efficacy over time.

Concomitantly with early impact on ulcer healing, the nurse‐led education program improved patient knowledge and self‐efficacy, as measured by the VeLUSET, with significantly higher scores in the IG compared to the CG at 1 month (261.6 (SD28) in IG versus 242.8 (SD39.1) in CG, *p* = 0.013) (Figure 4 and Table 2). Although a trend was observed, significant differences between groups were not observed at later time points.

**TABLE 2:** Impact of the nurse‐led education program on knowledge and health behaviours.

	Baseline			1 month			3 months			6 months			12 months		
	Intervention	Control	*p*	Intervention	Control	*p*	Intervention	Control	*p*	Intervention	Control	*p*	Intervention	Control	*p*
Knowledge ad self‐efficacy															
VeLUSET score, mean (SD) *p (intra) (ti* vs. *baseline)*	233.4 (41.7)	230.2 (43.7)	1.000	261.6 (28.0) **< 0.001**	242.8 (39.1) 0.176	0.013	264.9 (31.2) **< 0.001**	252.1 (37.8) **0.018**	0.092	267.7 (35.0) **< 0.001**	259.4 (27.2) **0.001**	0.214	271.4 (30.2) **< 0.001**	260.1 (26.9) **< 0.001**	0.070
Compression therapy															
Stockings, *n* (%) *p (intra) (ti* vs. *baseline)*	9 (19.6)	16 (39.0)	0.059	20 (43.5) **0.010**	12 (29.3) 0.423	0.189	29 (63.0) **< 0.001**	11 (26.8) 0.267	0.001	38 (82.6) **< 0.001**	26 (63.4) **0.044**	0.053	40 (87.0) **< 0.001**	34 (82.9) **< 0.001**	0.765
Bandages, *n* (%) *p (intra) (ti* vs. *baseline)*	42 (91.3)	30 (73.2)	**0.044**	34 (73.9) **0.013**	37 (90.2) 0.070	0.058	20 (43.5) **< 0.001**	29 (70.7) 1.000	0.017	13 (28.3) **< 0.001**	16 (39.0) **0.011**	0.364	12 (26.1) **< 0.001**	7 (17.1) **< 0.001**	0.436
Physical activity															
Physical activity, *n* (%) *p (intra) (ti* vs. *baseline)*	40 (87.0)	35 (85.4)	1.000	44 (95.7) 0.289	37 (90.2) 0.724	0.410	42 (91.3) 0.724	38 (92.7) 0.450	1.000	42 (91.3) 0.724	32 (78.0) 0.505	0.130	42 (91.3) 0.752	35 (85.4) 1.000	0.510
Minutes per day, mean (SD) *p (intra) (ti* vs. *baseline)*	55.2 (50.6)	61.1 (96.3)	0.450	68.7 (67.0) 0.545	44.4 (44.3) 0.946	0.147	66.6 (58.6) 0.336	48.6 (42.7) 0.474	0.108	70.6 (65.7) 0.278	60.2 (51.7) 0.216	0.510	105.6 (100.4) **0.017**	67.7 (87.8) 0.453	0.037
Ankle exercise, *n* (%) *p (intra) (ti* vs. *baseline)*	28 (60.9)	17 (41.5)	0.087	38 (82.6) **0.024**	29 (70.7) **0.003**	0.212	43 (93.5) **0.001**	28 (68.3) **0.015**	**0.004**	41 (89.1) **0.002**	32 (78.0) **0.001**	0.242	39 (84.8) **0.010**	33 (80.5) **0.001**	0.777
Minutes per day, mean (SD) *p (intra) (ti* vs *baseline)*	13.6 (14.4)	21.8 (32.2)	0.334	23.0 (23.4) **0.022**	23.4 (26.8) 0.289	0.944	30.1 (32.1) **< 0.001**	20.4 (18.1) **0.402**	0.110	23.9 (42.4) 0.175	23.9 (40.9) 0.170	0.998	35.7 (57.4) **0.005**	18.1 (16.8) 0.295	0.075
Leg elevation, *n* (%) *p (intra) (ti* vs. *baseline)*	37 (80.4)	39 (95.1)	0.054	40 (87.0) 0.505	36 (87.8) 0.248	1.000	44 (95.7) **0.023**	34 (82.9) 0.131	0.078	40 (87.0) 0.579	38 (92.7) 1.000	0.491	45 (97.8) **0.027**	37 (90.2) 0.683	0.183
Minutes per day, mean (SD) *p (intra) (ti* vs. *baseline)*	243.4 (198.3)	225.8 (218.8)	0.469	313.5 (224.7) 0.144	264.2 (203.5) 0.195	0.369	306.6 (192.6) 0.162	287.6 (214.1) 0.154	0.595	285.9 (204.9) 0.355	302.9 (185.6) 0.060	0.756	273.1 (201.8) 0.597	253.4 (198.4) 0.304	0.761
Accelerometry, mean (SD) Light (min) Moderate (min)	228.8 (194.0) 81.0 (75.0)	243.9 (205.6) 106.0 (85.9)	0.363 0.094	188.5 (163.0) 73.5 (68.4)	187.7 (89.4) 97.93 (82.9)	0.976 0.141	167.1 (86.6) 95.7 (90.5)	161.4 (56.2) 84.7 (71.2)	0.714 0.529	180.4 (94.2) 80.5 (74.7)	179.5 (70.2) 85.7 (65.7)	0.956 0.733	157.8 (66.1) 82.1 (62.2)	204.8 (138.9) 98.3 (71.8)	0.053 0.266
Nutrition															
MNA score at risk, *n* (%)	8 (17.4)	10 (24.4)	0.441	8 (17.4)	6 (14.6)	0.778	6 (13.0)	2 (4.9)	0.272	6 (13.0)	7 (17.1)	0.765	3 (6.5)	3 (7.3)	1.000
FFQ score, mean (SD)	0.76 (0.31)	0.78 (0.41)	1.000	0.83 (0.46)	0.8 (0.39)	0.741	0.86 (0.41)	0.8 (0.35)	0.484	0.82 (0.42)	0.73 (0.29)	0.266	0.78 (0.38)	0.77 (0.4)	0.933
FFQ score < 0.8, *n* (%)	30 (65.2)	24 (58.5)	0.522	29 (63.0)	22 (53.7)	0.375	34 (73.9)	25 (61.0)	0.197	32 (69.6)	26 (63.4)	0.544	36 (78.3)	28 (68.3)	0.293
BMI, mean (SD)	31.7 (9.0)	32.8 (9.0)	0.576	31.4 (8.8)	32.4 (8.4)	0.616	31.9 (9.1)	32.3 (8.6)	0.809	31.7 (9.0)	32.2 (8.3)	0.812	31.4 (9.6)	31.7 (10.2)	0.885

*Note:* Bold values indicate statistical significance at *p* < 0.05.

**FIGURE 5:**
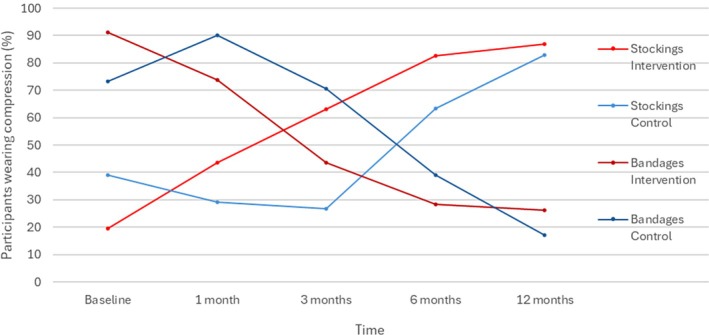
Adherence to compression therapy over time.

VeLUSET scores improved in both groups over the course of the follow‐up (Figure 5 and Table 2), reflecting the progressive acquisition of knowledge and self‐efficacy throughout the participants' care trajectory and serving as an indicator of care quality. It is to be noted that this improvement in VeLUSET scores occurred more quickly among participants who attended the educational sessions, as it was statistically significant after 1 month, whereas it became significant after 3 months for participants allocated to standard of care.

#### 3.3.2 Adherence to Compression Therapy

As compression therapy evolved throughout the care process, adherence was assessed in terms of bandage and stocking use. At 3 months, a significantly higher proportion of participants in the intervention group (IG) were wearing compression stockings compared to the control group (CG) (63%, *n* = 29/46 vs. 26.8%, *n* = 11/41; *p* = 0.001), despite a lower baseline rate in the IG (19.6% vs. 39%; *p* = 0.059). Conversely, bandage use at 3 months was significantly lower in the IG (43.5%, *n* = 20/46) than in the CG (70.7%, *n* = 29/41; *p* = 0.017) (Table 2). While similar trends persisted at later time points, group differences diminished by 12 months.

Importantly, the transition from bandages to stockings occurred earlier in the IG—by 3 months compared to 6 months in the CG (Figure 5 and Table 2). As with the VeLUSET score, adherence to compression therapy improved progressively in both groups, suggesting a general behaviour change over time. However, the shift was notably faster among participants in the education program: stocking use increased significantly by 1 month in the IG, compared to 6 months in the CG. Similarly, bandage use declined earlier in the IG, reinforcing the short‐term impact of structured education.

#### 3.3.3 Physical Activity

##### 3.3.3.1 Practice of Physical Activity

The proportion of participants reporting daily physical activity (e.g., a minimum 10‐min walk) was similar in both groups at baseline (87.0%, *n* = 40/46 in IG; 85.4%, *n* = 35/41 in CG) and remained stable throughout the study (Table 2). In the IG, the mean time dedicated to physical activity increased from 55.2 min (SD 50.6) at baseline to 105.6 min (SD 100.4) at 12 months (*p* ^*intra*^ = 0.017). No significant change was observed in the CG (61.1 min, SD 96.3 at baseline vs. 67.7 min, SD 87.8 at 12 months *p* ^*intra*^ = 0.453) (*p* = 0.037) with a significant difference between groups (*p* = 0.037).

Objective activity data collected using GeneActiv accelerometers over 1 week at each timepoint showed no difference in average daily time spent in light and moderate intensity activity between groups, and no change over time (Table 2).

##### 3.3.3.2 Practice of Ankle Exercises

The proportion of participants reporting to practice targeted exercises of the ankle was moderate (60.9%, 28/46, in IG and 41.5%, *n* = 17/41, in CG, *p* = 0.111) at baseline and increased significantly throughout the study in both groups (Table 2). At 3 months, it was significantly higher in the IG versus CG (93.5%, *n* = 43/46, in IG vs. 68.3%, *n* = 28/41, in CG, *p* = 0.004), but this difference diminished during the study. Participants attending educative sessions significantly increased the time they dedicated to this activity during the study (mean time in minutes: 13.6 (SD 14.4) at baseline to 35.7 (SD 57.4) at 12 months, *p* ^*intra*^ = 0.005) while this was not the case in CG (21.8 (SD 32.2) at baseline to 18.1 (SD 116.8) at 12 months, *p* = 0.295) (*p* = 0.075).

##### 3.3.3.3 Practice of Leg Elevation

In both groups, the majority of participants reported elevating their legs at the beginning of the study. This proportion remained high until the end of the follow‐up in both groups, with a significant upward trend among participants receiving the nurse‐led educative sessions (80.4% at baseline to 97.8% at 12 months in the IG, *p* = 0.027; 95.1% at baseline to 90.2% at 12 months in the CG, *p* = 0.683) (difference between the two groups at 12 months: *p* = 0.183). The time that participants spent elevating their legs did not change throughout the study (Table 2).

#### 3.3.4 Nutrition

##### 3.3.4.1 Risk of Malnutrition

The MNA indicated that a minority of participants were at risk of malnutrition at baseline (17.4%, *n* = 8/46, in IG and 24.4%, *n* = 10/41, in CG, *p* = 0.590). These percentages tended to decrease during the study in both groups, but the nurse‐led therapeutic education programme did not significantly impact this reduction (Table 2).

##### 3.3.4.2 Protein Intake

Nearly 60% of all participants were consuming protein amounts below the recommended level of 0.8 g/kg/d at baseline (65.2%, *n* = 30/46, in IG and 58.5%, *n* = 24/41, in CG, *p* = 0.522), with a mean FFQ score of 0.76 (SD 0.31) in IG and 0.78 (SD 0.41) in CG (*p* = 0.522). The program did not result in significant changes in protein intake per kg body weight per day within or between groups.

##### 3.3.4.3 Body Weight Control

At baseline, the mean BMI in both groups was markedly elevated, with values exceeding 30 kg/m^2^ (intervention group: 31.7 ± 9.0; control group: 32.8 ± 9.0; *p* = 0.576). The intervention did not produce a significant effect on weight reduction, as BMI values remained consistently high across both groups throughout the study period (see Table 2).

## 4 Discussion

The aim of this study was to evaluate the impact of a multidisciplinary nurse‐led education program on VLU healing, patient knowledge acquisition, and engagement in health behaviour. The study population closely aligns with previous research, where VLU patients are predominantly older adults with obesity and chronic venous insufficiency [28, 29, 30, 31, 32]. Recurrent ulcers were again similar to other studies at 65.2% IG, 61% CG [9, 33], where recurrence rates ranged from 57% to 73% over 1–2 years. Compared to previous studies, our mean age of 68.2 years aligns with other research [33, 34], which reported VLU prevalence peaking in patients over 60. Obesity, defined by BMI values above 30, was also a key characteristic of the study population (mean BMI 32.2), consistent with Rosenburg et al. [35]. Common comorbidities such as diabetes were observed, and these findings were similar to other published research [14], emphasising their role in delayed healing and increasing the risk of recurrence.

The education program accelerated wound healing, with 17.4% of ulcers healed in the IG at one month vs. 2.4% in the CG (*p* = 0.032), and 39.1% vs. 31.7% at three months (*p* = 0.508). This aligns with a recent systematic review and meta‐analysis [14] that reported education improves patient engagement, leading to faster healing. The PWAR was significantly higher in the IG at one month (54% vs. 35.6%, *p* = 0.020), with more patients achieving ≥ 40% wound reduction (67.4% vs. 46.3%, *p* = 0.047), a known predictor of healing [20]. Similar findings in our pilot study [19] and other research [6] confirm that education enhances healing when combined with compression therapy. As highlighted by He et al. [9], adherence to self‐management is key to healing, reinforcing that structured education plays an important role in VLU treatment.

Our study has gone beyond the predominant practice of assessing healing outcomes at3 to 66 months and had a study duration of 12 months [36, 37]. Our results raised some important points that should be considered in future RCTs. At one and three months, the intensive education intervention showed improved healing outcomes and a greater reduction in wound size, both of which are commendable. But, this was not sustained once the intervention was reduced to phone consultations rather than one‐to‐one education sessions. It shows that had we closed the trial at 3 months, we would have concluded that the intervention had superior outcomes to control, but through longer follow‐up, this was not sustained, and thus we avoided a Type 1 error. It raised the possibility that any intervention may need redesign at 3 months in order to reboot the healing response rate and the value of having a longer duration for any trial. These short‐term benefits could be directly linked to the temporal structure of the intervention, which offers very frequent educational sessions during the first month of the program and much fewer, more spaced‐out sessions in the following months. This would suggest that maintaining improvements may require a program whose educational sessions would remain closely spaced over a longer period. Alternatively, this rapid effect could also reflect the impact of the intervention on a subset of wounds with a higher healing potential due to the patient's health context.

The multidisciplinary education program not only accelerated wound healing in the early stages but also improved patient knowledge, compression therapy adherence, and mobility, although its impact on nutrition was limited. Knowledge and self‐efficacy significantly improved within 1 month (VeLUSET score: 261.6 vs. 242.8, *p* = 0.013). Compression therapy adherence increased at 3 months (63% vs. 26.8%, *p* = 0.001), with faster transitions from bandages to stockings. This supports findings from Bar et al. [11], who reported higher adherence following structured education. Behairy and Masry [15] also described that nurse‐led education improves the use of compression therapy and reduces recurrence risk.

Mobility showed notable progress, with participants in the intervention group increasing their daily activity (55.2–105.6 min, *p* = 0.017) and their daily practice of ankle exercises (13.6–35.7 min, *p* = 0.005), whereas no significant change was seen in the control group. This finding aligns with evidence that higher levels of physical activity enhance venous return, strengthening the calf muscle pump and reducing ulcer recurrence [33, 34].

Despite improvements in other areas and similar to other research, nutritional changes were minimal [38]. Although there was a slight decline in malnutrition risk in both groups (17.4% at baseline to 6.5% at 12 months in IG, *p* = 0.131 and 24.4% to 7.3% in CG, *p* = 0.070), protein intake remained insufficient, and BMI remained high for most participants. Limited dietary progress is often linked to financial constraints and lack of awareness, indicating that education alone may not be enough to drive long‐term nutritional improvements [9]. A significant change in eating habits proves to be difficult to achieve and would likely require regular follow‐up by a dietitian. Although a dietitian was part of the multidisciplinary program, the limited impact on nutritional behaviour suggests that more intensive or sustained dietitian‐led interventions, such as individualised counselling or financial support, may be required to achieve long‐term dietary improvements. Structured nutritional counselling has been shown to support patients in modifying eating habits more effectively [39].

## 5 Strengths and Limitations

This study's multidisciplinary approach, integrating nursing, physiotherapy, and dietetics, provided a comprehensive educational intervention, improving patient knowledge, adherence to compression therapy, mobility, and wound healing. The randomised controlled design minimised selection bias, and the multiple sites strengthened real‐world applicability.

A key strength of this study was the 12‐month follow‐up and shows that early benefits of an intervention are not necessarily maintained over time. Although the final sample size was small, it is nonetheless reflective of many trials in the management of VLUs [36].

However, several biases may have influenced results. Selection bias was reduced through randomisation, but the small sample size (*n* = 87) and COVID‐19 recruitment challenges limit generalisability, necessitating larger trials. Differences in clinical practices between recruitment sites may also have had an impact on the observed results. Recall bias in self‐reported adherence to compression therapy, mobility, and nutrition may have overestimated behaviour changes; future studies should use wearable monitors or digital tracking for long periods of time. Observer bias may have affected wound assessments, as study nurses were unblinded due to the nature of the intervention. Although a different nurse was assigned to each study group, this issue suggests a need for independent assessors. This was addressed by blinding of a wound expert and dermatologist performing wound tracing verification, as well as blinding of the statistician conducting the analyses. Attrition bias due to dropout rates (28.3% intervention, 17.1% control) was addressed with multiple imputation. Lastly, the study focused on short‐term outcomes (12 months), whereas long‐term behaviour changes and recurrence prevention require further exploration.

## 6 Conclusion

A nurse‐led, multidisciplinary education programme improved early wound healing, patient knowledge, and adherence to compression therapy and mobility in individuals with VLUs. These effects were most evident in the first 3 months but declined over time, suggesting that sustained support is needed to maintain behavioural change. Nutritional outcomes remained unchanged, indicating the need for more intensive, personalised dietary interventions. Future research should explore long‐term education models, digital follow‐up strategies, and tailored nutritional support to optimise self‐management and reduce recurrence in VLU care.

## Conflicts of Interest

The authors declare no conflicts of interest.

## Data Availability

The datasets generated and analyzed during the current study are available upon reasonable request from the corresponding author. Upon publication, the data will also be made accessible through the Yareta repository at https://yareta.unige.ch.
